# Supplementary material for: Genome-Wide Identification of the TIFY Family in Cannabis sativa L. and Its Potential Functional Analysis in Response to Alkaline Stress and in Cannabinoid Metabolism
Source: Int J Mol Sci. 2025 Aug 22;26(17):8171. doi: 10.3390/ijms26178171 (PMC12428757; doi:10.3390/ijms26178171)
Supplement: Supplementary file 1 [file ijms-26-08171-s001.zip › Supplementary Data.pdf]

Supplementary Figure S1. Experimental flowchart.

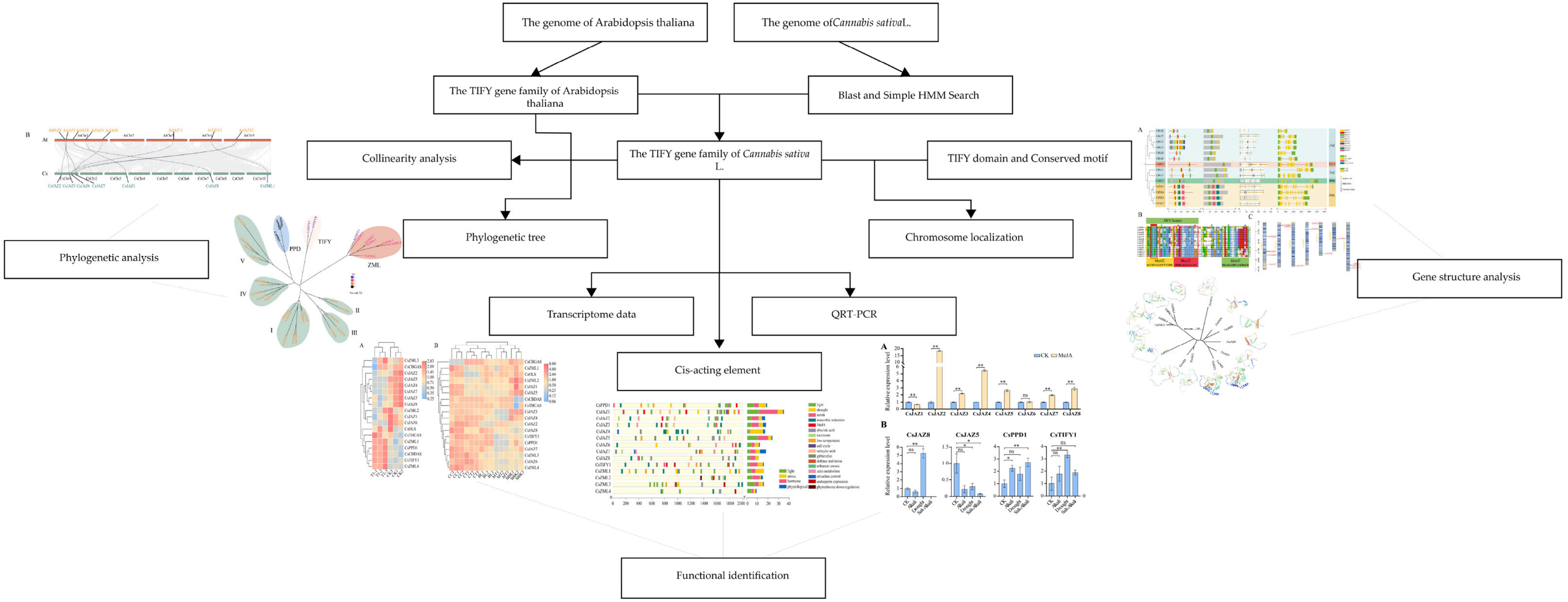

Supplementary Table S1. The information of 5 transcriptome data.

ID	PRJNA672722
Description	To obtain insights into the molecular mechanisms of alkaline salt tolerance, the transcriptome analysis of roots of industrial hemp was performed to identify the genes associated with the alkali tolerance. This is significant for the industrial hemp breeding and improvement of industrial hemp yield and quality of alkali-resistant industrial hemp
SRA	SRX9428679、SRX9428680、SRX9428681、SRX9428682、SRX9428683、SRX9428684、SRX9428685、SRX9428686、SRX9428687、SRX9428688、SRX9428689、SRX9428690
ID	PRJNA498707
Description	Glandular trichomes were mechanically removed from late-stage flowering plants of female cannabis inflorescence.Transcriptome sequencing was performed on the glandular hair tissue.
SRA	SRX5001683、SRX5001684、SRX5001685、SRX5001686、SRX5001687、SRX5001688、SRX5001689、SRX5001690、SRX5001691、SRX5001692、SRX5001693、SRX5001694、SRX5001695、SRX5001696、SRX5001697、SRX5001698、SRX5001699、SRX5001700、SRX5001701、SRX5001702、SRX5001703、SRX5001704、SRX5001705、SRX5001706、SRX5001707、SRX5001708、SRX5001709
ID	PRJNA756306
Description	The objective in this study was to develop and characterize the chemistry and genetics of a collection of feral hemp germplasm from across Nebraska for use in a hemp breeding program. An additional goal was to compare the chemistry and genetics of male and female flower structures. RNA was extracted from isolated, dissected flower tissue of one female and one male plant from each of three populations (total six samples). These plants were derived from seeds collected in Knox, Madison, and Merrick counties. Illumina reads were obtained from the RNA extracts, mapped onto a C. sativa reference genome, and gene expression levels were determined. Overall design: mRNAs in female and male flower tissues

SRA	SRX11828417、SRX11828418、SRX11828419、SRX11828420、SRX11828421、SRX11828422
ID	PRJNA1108719
Description	Cannabis sativa L., which has been reclassified as an agronomic crop, has experienced an increase in cultivation. Its interactions with a variety of environmental stressors have been extensively studied. However, the mechanisms of recovery through fungal associations remain underexplored. Trichoderma hamatum, known for its role as a biological agent, enhances plant growth and provides antagonistic defense against pathogenic microbes. This meta-dataset aims to investigate whether Th can enhance drought resistance in a Cannabis plants. Overall design: To investigate how Trichoderma hamatum treatment manifests drought resistance mediation activities at the transcriptome level in Cannabis sativa L., adult Cannabis plants were used. Drought treatment was achieved by halting irrigation, a stress method that hemp can experience in field cultivation. Trichoderma hamatum was isolated from the soil of the cannabis cultivation area using Trichoderma Selective Medium, and then separated into a single fungal strain through the hyphal tip separation process. The fungal strain's TEF1, ITS, and Large Subunit rRNA were sequenced, and identified using NCBI BLASTn. The experimental groups were designed under four different conditions: control, drought stress, T. hamatum single treatment, and a combination of drought stress and T. hamatum treatment.
SRA	SRX24485620、SRX24485621、SRX24485622、SRX24485623、SRX24485624、SRX24485625、SRX24485626、SRX24485627、SRX24485628、SRX24485629、SRX24485630、SRX24485631

ID	PRJNA1199007
Description	Known to infect more than 600 plant species worldwide, Sclerotinia sclerotiorum is a necrotrophic fungal pathogen, and the causative agent of white mold. With recent infection reports documented across North America, Cannabis sativa is known to be susceptible to Sclerotinia infection. Resulting from legal constraints associated with C. sativa, little is known about the Cannabis-Sclerotinia pathosystem, particularly in how the plant responds to pathogen attack at the cellular and molecular levels. Anatomical study revealed initial infection and degradation of the epidermis and cortical parenchyma, followed by widespread infection of the vascular phloem. Dual RNA sequencing provided a detailed transcriptomic profile of this pathosystem directly at the site of infection. Differential gene expression analysis revealed large-scale transcriptional shifts resulting from rapid infection. We identified the upregulation of 97 genes at 1 day post inoculation (dpi) and 6733 genes 5 dpi in C. sativa, while 3186 genes were identified in S. sclerotiorum 7 dpi. Gene ontology term enrichment identified processes associated with plant defense and signal transduction cascades during C. sativa infection while processes associated with redox control and sugar catabolism were enriched in S. sclerotiorum. Taken together, this study revealed transcriptional reprogramming in both the host plant and fungal pathogen associated with degradation of host cortical and vascular phloem tissues. Overall design: Cannabis sativa (cultivar 'Kona') inflorescences were inoculated with Sclerotinia sclerotiorum. Infected and uninfected tissues were collected at 1 day post inoculation (dpi), 3 dpi, 5 dpi and 7 dpi
SRA	SRX27102495、SRX27102496、SRX27102497、SRX27102498、SRX27102499、SRX27102500、SRX27102501、SRX27102502、SRX27102503、SRX27102504、SRX27102505、SRX27102506、SRX27102507、SRX27102508、SRX27102509、SRX27102510、SRX27102511、SRX27102512、SRX27102513、SRX27102514、SRX27102515、SRX27102516、SRX27102517、SRX27102518、SRX27102519、SRX27102520、SRX27102521、SRX27102522、SRX27102523、SRX27102524、SRX27102525、SRX27102526

Supplementary Table S2. QRT-PCR primers for 14 pairs of CsTIFYs.

Primer name	5'to 3'
EF1a-100F	TGTTTTGCACGGATCAGTTTG
EF1a-100R	AATGCCGACCGCTACAGTTC
CsJAZ1-F	CCCAAGTCCTCTAGTGCACCT
CsJAZ1-R	AACCATTTCCAGCCAGTAACAT
CsJAZ2-F	GGCAAAGAGCAACAACGCA
CsJAZ2-R	ACTCTGGGATTTGGTATGGTGC
CsJAZ3-F	ACCAGGCTAAGAGCAACAACG
CsJAZ3-R	GCTAATTCTGGGATTTGGTATGG
CsJAZ4-F	CCTTCCTGAAACAACTCCATTAACT
CsJAZ4-R	GTTTGAAAGAGGTGGTGTTGGAT
CsJAZ5-F	TCAAGGTCGTTGTCGGTCTCT
CsJAZ5-R	GGTCTACTTTGCTCACAACTGCTT
CsJAZ6-F	ATGGTGGGTGCGTCAAGAT
CsJAZ6-R	CGGAGGAACTGGTGAACTAGAG
CsJAZ7-F	CCGATTGATAAAGCGAACGA
CsJAZ7-R	GACTTCATCAGTGTCAGCAGGAG
CsJAZ8-F	GCAAGTAGAGAAATGGAGGAGAGA
CsJAZ8-R	CGTTGGGCTGTAAATCGGT
CsTIFY1-F	GGAGACAGACCGAGAAGAATGA
CsTIFY1-R	TTGAGGCACCATAGGCACAA
CsPPD1-F	GCAACTAGCAGCAAGTCCAAT
CsPPD1-R	ACACTAGGAGGAACACCACATT
CsZML1-F	TAATCCTCACCTCCGCTACG
CsZML1-R	TCCTCAACGACACCTCCAC
CsZML2-F	GTGTCCAGGCTGCTTCAATG
CsZML2-R	AGACGATGTTGGTTGTTGTGTT
CsZML3-F	CTGCCAATCCACAACCTCTG
CsZML3-R	ACGACCATCTTCATACTCATCTTC
CsZML4-F	GAGACCGTATGTAGCAGAAGGA
CsZML4-R	TCCACCTCCACCTCCAACA
CsTHCAS-F	GCTCACTTAGTCAACGCTGAT
CsTHCAS-R	CTTCACAGTCTCATTCATCTCCAA
CsCBDAS-F	GGTCTTGGAGGTGATAATGTTGTA
CsCBDAS-R	CAGTGGCTATTGTCAATGAAGATG
CsOLS-F	CAGTGAAGCGTGTGATGATGT
CsOLS-R	ACAACAGACGGCGAGAACT
CsCBGAS-F	GAGCATAATTGTGGCACTAACTG
CsCBGAS-R	TCTAATTGGTGGAACAGAATAGGC

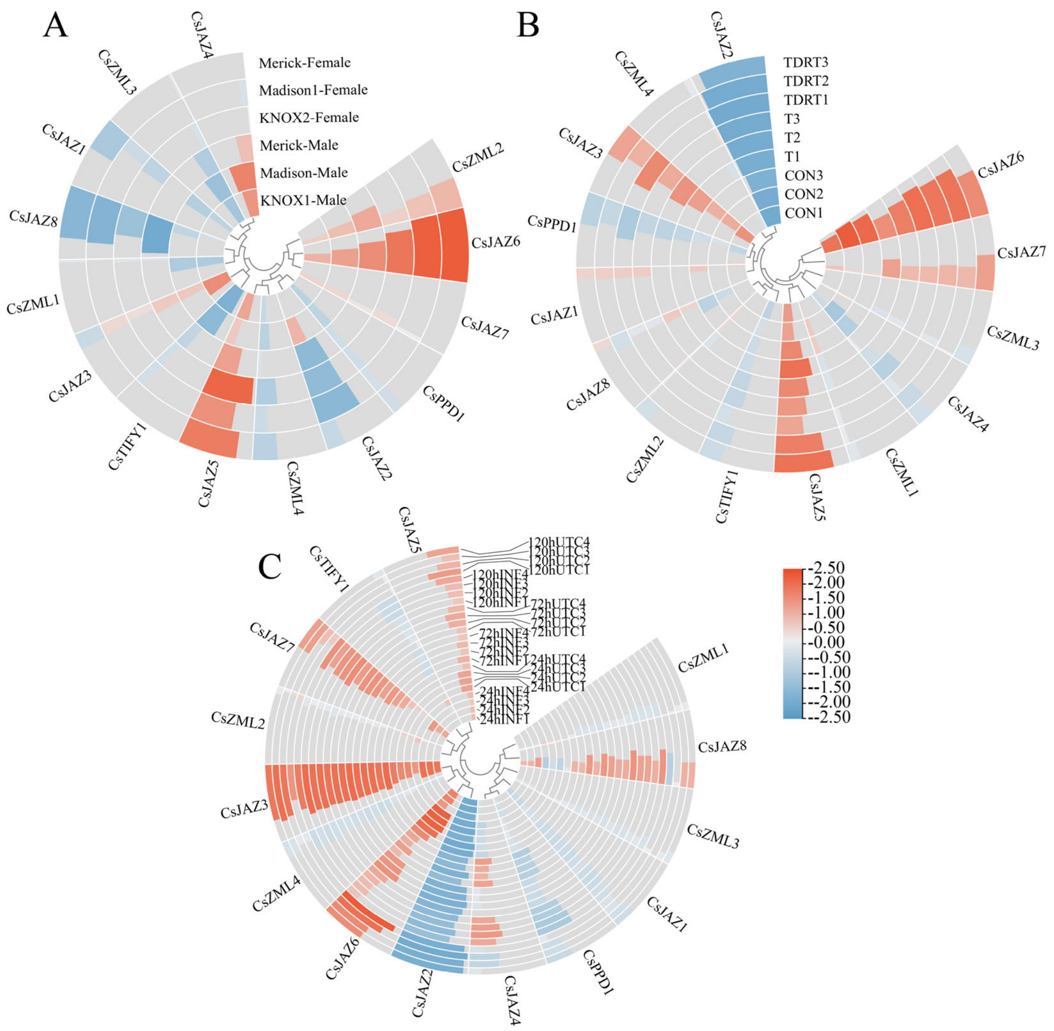

Supplementary Figure S2. Gender difference and Stress expression heatmap of TIFY in C. sativa. A: Differences in TIFY of C. sativa across different sexes. 3 wild C. sativa: Merick, Madison, KNOX. B: Differential Expression of TIFY in C. sativa under alkali stress. T – CK1 – 3: Control group (0mmol/L NaHCO3); T – 1 – 3: Alkali treatment group (100mmol/L). C: Differential Expression of TIFY in C. sativa under drought stress and during recovery mediated by fungal symbiosis. CON1 – 3: Control group; T1 – 3: Trichoderma-treated group; TDRT1 – 3: combined drought + Trichoderma treatment group. D: Differential expression of TIFY in C. sativa during Sclerotinia sclerotiorum infection. 24hINF1 – 4: Infection group (24 hours); 24hUTC1 – 4: Uninfected group (24 hours); 72hINF1 – 4: Infection group (72 hours); 72hUTC1 – 4: Uninfected group (72 hours); 120hINF1 – 4: Infection group (120 hours); 120hUTC1 – 4: Uninfected group (120 hours).

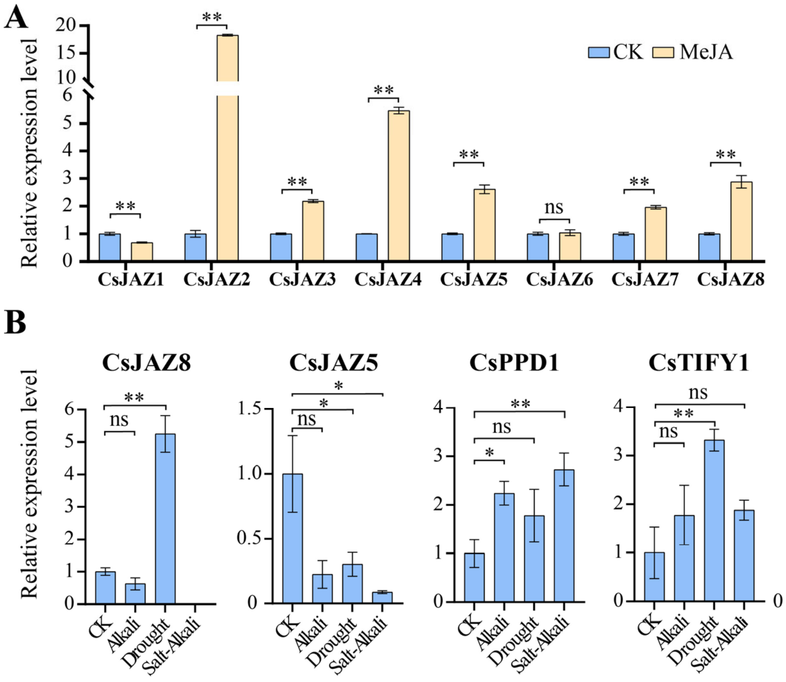

Supplementary Figure S3. Relative expression levels of TIFY in C. sativa. under different treatments. A: Relative expression level of CsJAZs after 24 h of treatment with 50 mmol/L MeJA. B: Relative expression levels of CsJAZ5/8, CsPPD1, and CsTIFY1 under alkali stress, drought stress and salt-alkaline stress, respectively.

Supplementary Table S3. The numerical value of the cannabinoid content.

Cannabinoids	BBK	BL	CT	CC	MT
THCA	13.56±0.90	15.02±1.10	3.19±0.20	16.55±0.81	5.91±0.60
THC	0.31±0.02	1.62±0.19	0.55±0.055	0.15±0.008	0.14±0.02
CBDA	0.45±0.02	0.12±0.012	7.76±0.63	0.079±0.007	0.016±0.003
CBD	0.95±0.07	0.139±0.016	0.085±0.013	0.079±0.008	0.047±0.005
Cannabigerol	0.12±0.015	0.086±0.008	0.093±0.008	0.051±0.005	0.016±0.001
Cannabinol	1.74±0.20	0.55±0.019	0.53±0.051	0.83±0.019	0.29±0.028
Total cannabinoids	15.87±1.13	17.53±1.25	12.20±0.85	17.74±0.82	6.41±0.65

Supplementary Table S1 is quoted from "Zager JJ, Lange I, Srividya N, Smith A, Lange BM. Gene Networks Underlying Cannabinoid and Terpenoid Accumulation in Cannabis. Plant Physiol. 2019 Aug;180(4):1877-1897.".

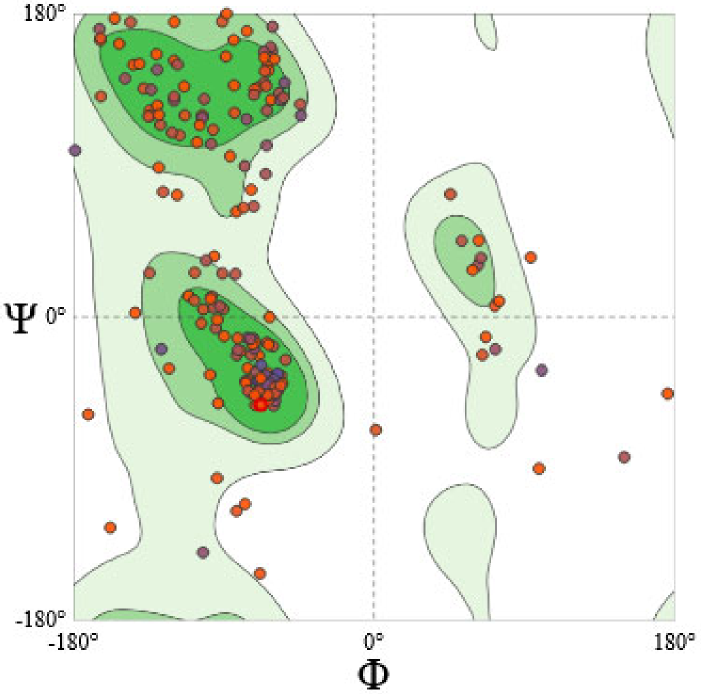

Supplementary Figure S4. Ramachandran favorable plot of CsJAZ2.

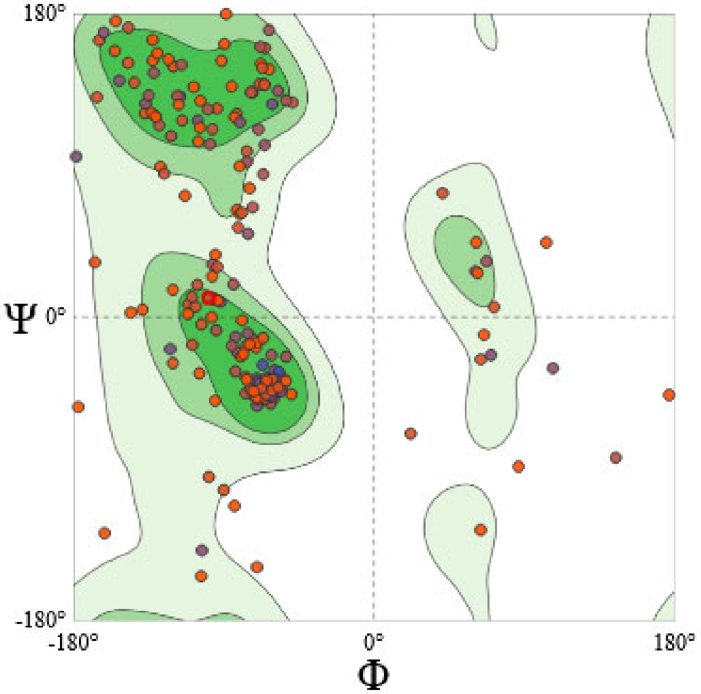

Supplementary Figure S5. Ramachandran favorable plot of CsJAZ3.
